# Supplementary figures and images for: The endoplasmic reticulum promotes microtubule organization and region-specific disassembly to execute Compartmentalized Cell Elimination
Source: bioRxiv. 2025 May 13:2025.05.08.652974. Preprint. [Version 1] doi: 10.1101/2025.05.08.652974 (PMC12132283; doi:10.1101/2025.05.08.652974)

S1A

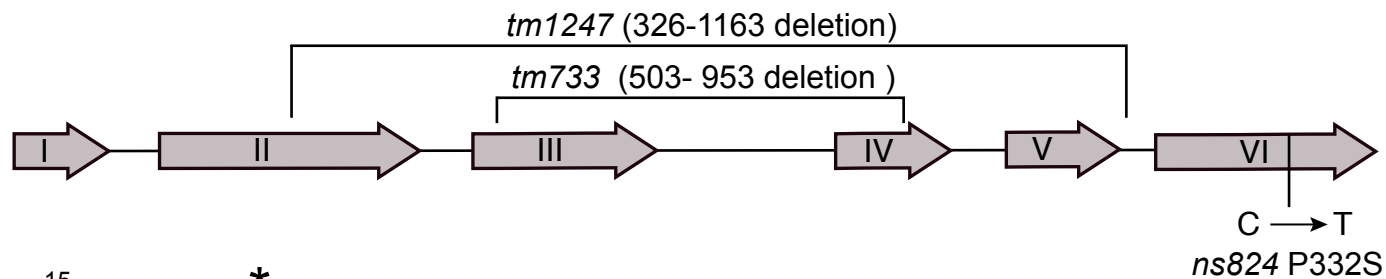

S1B

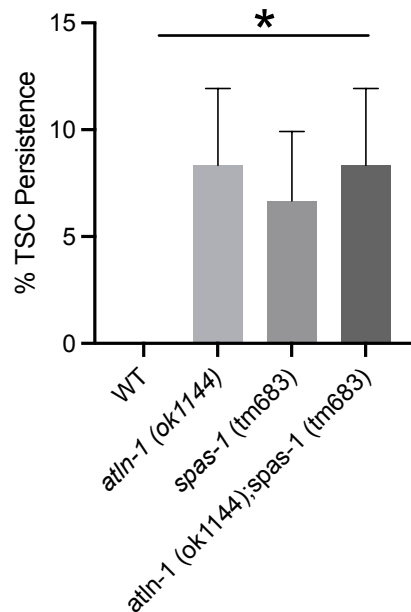

Supplement: Supplement 1 — Supplemental Figure S1 lnp-1 gene structure showing the allele location and double mutant graph. [file media-1.pdf]

## TBA-1

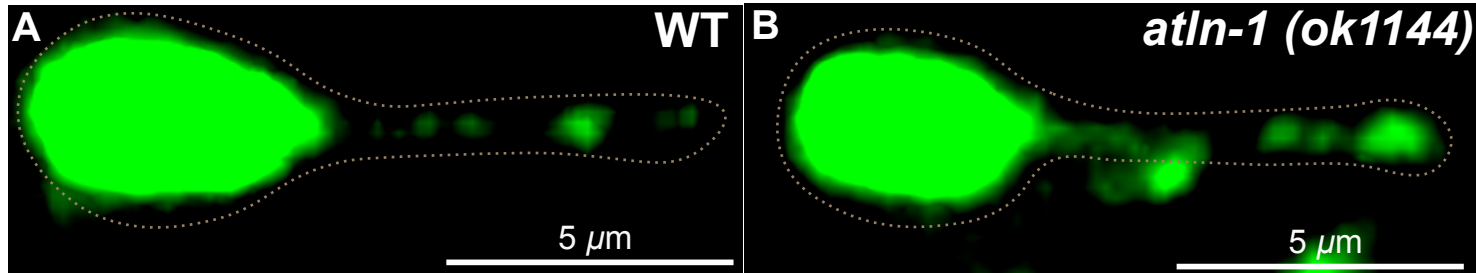

Supplement: Supplement 2 — Supplemental Figure S2 Co-labeling TBA-1 and SPAS-1 in soma distal degrading (SDD) in wildtype and atln-1(ok1144) to show distal tip GFP signal. TBA-1 signal in wildtype distal tip appears smaller and discrete. Where in atln-1(ok1144) it appears larger and less distributed. [file media-2.pdf]

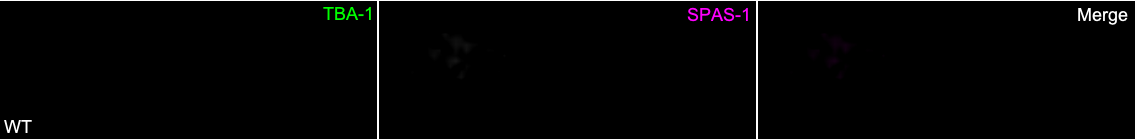

Supplement: Supplement 4 [file media-4.tif]

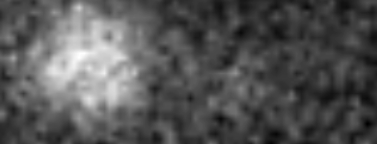

Supplement: Supplement 5 [file media-5.tif]
