# Supplementary material for: The endoplasmic reticulum promotes microtubule organization and region-specific disassembly to execute Compartmentalized Cell Elimination
Source: bioRxiv. 2025 May 13:2025.05.08.652974. Preprint. [Version 1] doi: 10.1101/2025.05.08.652974 (PMC12132283; doi:10.1101/2025.05.08.652974)
Supplement: 6 [file NIHPP2025.05.08.652974v1-supplement-6.pdf]

bioRxiv preprint doi: https://doi.org/10.1101/2025.05.08.652974; this version posted May 13, 2025. The copyright holder for this preprint (which was not certified by peer review) is the author/funder. This article is a US Government work. It is not subject to copyright under 17 USC 105 and is also made available for use under a CC0 license.

Supplemental Tables

Supplemental table 1 Plasmid

Supplemental table 2 Transgenes and strains

Supplemental Movies

Supplemental movie M1 Co-labeling TBA-1 and SPAS-1 in soma distal degrading (SDD) in wildtype embryos during CCE. Movies show planes across the TSC.

Supplemental movie M2 Co-labeling TBA-1 and SPAS-1 in soma distal degrading (SDD) in *atln-1(ok1144)* embryos during CCE. Movies show planes across the TSC.
